# Evaluation of the effects of addiction levels and physical activity capacities of smokers on exhaled carbon monoxide level

**DOI:** 10.1017/S1463423625100108

**Published:** 2025-05-29

**Authors:** Ecem Çakir Altinyaprak, İzzet Fidanci, Fatma Birgül Kumbaroğlu, Tülin Düger

**Affiliations:** 1 Faculty of Medicine, Department of Family Medicine, Hacettepe University, Ankara, Turkey; 2 Faculty of Physical Therapy and Rehabilitation, Hacettepe University, Ankara, Turkey

**Keywords:** Carbon monoxide, nicotine dependence, physical activity, smoking

## Abstract

**Aim:**

This study aims to evaluate the effect of smokers’ nicotine addiction levels and physical activity capacities (aerobic capacities) on exhaled carbon monoxide (CO) measurement values in respiratory air.

**Methods:**

This study is a cross-sectional epidemiological descriptive type study. About 146 smokers, aged 18 and above, who applied to Hacettepe University Family Medicine outpatient clinics between March and May 2023 were included in the study. The Fagerström Test for Nicotine Dependence (FTND) and 6-minute walk test (6-MWT) were performed to the participants, and the relationship of the data with exhaled CO level was examined.

**Results:**

In the data we obtained, it was observed that the addiction score evaluated by the FTND had a positive, moderately statistically significant effect on the CO level (r = 0.483 p < 0.001). Although the percentage of aerobic capacity (physical activity capacities) assessed by the 6-MWT appeared to have a very weak negative relationship with the exhaled CO level, it was found to be not statistically significant (r = −0.112 p = 0.177).

**Conclusion:**

The data we obtained showed that smoking addiction has harmful effects such as increasing CO in the body, but there is no relationship between physical activity and the amount of exhaled CO. It has been observed that quitting smoking and complying with lifestyle change recommendations are an important necessity for a healthier life. To improve patients’ overall health outcomes, family physicians are crucial in helping patients quit smoking and encouraging lifestyle modifications. This study might have encouraged the reflection of smoking habits and thus motivated quitting.

## Introduction

Tobacco is defined by the World Health Organization (WHO) as ‘a psychoactive substance that causes mental and behavioural disorders’ and causes many physical diseases and high health care costs (Raw *et al.*, 2002). According to WHO data, 19.9% of the total world population, 6.2% of the female population, and 33.7% of the male population are smokers (WHO, 2019). Nicotine in tobacco causes tobacco addiction, which requires biological and behavioural treatment (Örsel, 2010). Tobacco addiction has been recognized as a chronic disease, and it is important to evaluate the level of nicotine in tobacco to determine the appropriate treatment for smoking cessation (Tobacco Use and Dependence Clinical Practice Guideline, 2000; Fiore *et al.*, 2008). Physical activity is an important element in protecting and maintaining health. Regular physical activity can prevent many diseases and improve the quality of life (Görek Dilektaşlı, 2019; Şen, 2017).

In this study, we aimed to evaluate the effect of nicotine dependence levels and physical activity capacities on exhaled carbon monoxide (CO) measurement values in respiratory air in smokers who applied to our family medicine clinics.

## Methods

This study is a cross-sectional epidemiologic descriptive study. It was conducted at Hacettepe University, Faculty of Medicine, Department of Family Medicine. Ethical approval of the study was granted on 05.07.2022 by Hacettepe University 2022/12 Meeting of the Non-Interventional Clinical Research Ethics Committee Number, Decision number 2022/12-51 and GO 22/690 Research/Project Received by number. The data collection part of the study was completed between 28.03.2023-23.05.2023. Between 23.05.2023 and 22.10.2023, data analysis and reporting stages were completed. This study was conducted in accordance with the ethical principles outlined in the Declaration of Helsinki, ensuring the protection of human subjects involved in research. The population of the study consisted of participants aged 18 and above who applied to the Family Medicine Outpatient Clinic of Hacettepe University Faculty of Medicine, who smoked, agreed to participate in the study, signed the informed consent form, and whose CO levels were previously measured in family medicine outpatient clinics. Cancer patients, pregnant or breastfeeding women, patients with psychiatric disorders, patients with orthopaedic or neurological diseases that may affect walking, patients with diseases that may change lung capacity, those with incomplete or inconsistent data, and those who did not accept the informed consent form were excluded from the study. There was a total of 146 participants in the study and 7 individuals (2.2%) declined to participate in the study. However, no participants dropped out or failed to complete the study after agreeing to participate. Those who declined to participate were homogeneous with the study participants and had similar mean ages.

The participants’ sociodemographic characteristics such as age, gender, occupation, education status, marital status, employment status, amount of smoking, and regular exercise status were recorded with the help of an 8-question questionnaire. Afterwards, the participants’ addiction levels were measured with the 6-question Fagerstrom Test for Nicotine Dependence (FTND), and their physical activity levels were evaluated with the 6-minute walk test (6-MWT) performed by a physiotherapist.

### 6-minute walk test

6-MWT is a field test that provides information about aerobic capacity according to the distance patients can walk at a normal pace for six minutes in a straight corridor without running (Görek Dilektaşlı, 2019). In an ideal environment, this test is performed in a 30-meter corridor; however, the distance can be changed according to the environmental conditions. In our study, this test was performed by the same physiotherapist on a flat surface in a hospital corridor, where the lap distance was determined to be 21 meters, and the same verbal commands recommended by the American Thoracic Society were applied to all subjects. 6-MWT distance was calculated as (number of laps x 21) + additional distance walked. Before 6-MWT, blood pressure was measured with a CE certified device (Blood pressure monitor, BM27, Beurer Medical, Germany). In all subjects, oxygen saturation was measured with a CE-certified pulse oximeter (Pulse oximeter, 12-1926, Beijing Choice Electronic Technology, China) and the pulse rate was measured before, during, and at the end of the test. The expected 6-MWT distance of all subjects was calculated according to the Enright–Sherrill Formula (Enright and Sherrill, 1998) using age, gender, and body mass index. There were no subjects who did not complete the test in the study.

### Measurement of exhaled CO

Exhaled CO measurement is commonly known as a breath test, and this test measures CO levels from a person’s breathing air. The measurement of exhaled CO levels is much more objective and reliable in assessing nicotine intake than questioning the amount and duration of smoking (Wan Puteh *et al.*, 2023; Sönmez *et al.*, 2017; Tabataba, 2023). In smokers, exhaled CO measurement assesses the combination of CO produced by the person’s body and CO taken in by smoking. The meter shows the level of exhaled CO in a person’s breath in ppm (parts per million), which is the number of CO molecules in one million air particles.

The exhaled CO ppm measurement can vary depending on the person’s smoking habit and how long it has been since the last cigarette (0 – 400pm). Exhaled CO levels are generally seen in the range of 0 – 6 ppm in normal non-smokers, and these values may increase with increased cigarette consumption. High CO levels, which can be easily detected by exhaled CO level measurement, generally indicate active smoking and increased health risks. The device we use (TABATABA CO-Tester®) measures CO based on a chemical reaction. Inside the electrochemical sensor is a special electrochemical cell that reacts with CO gas and CO levels are calculated by measuring the electrical changes during the reaction (Tabataba, 2023).

In the outpatient clinic, the patient is first asked to exhale completely, then to hold his/her breath for 15 seconds after taking a deep breath and slowly exhale into the exchangeable mouthpiece. The measured CO level is recorded.

### Fagerstrom Test for Nicotine Dependence

The FTND is a questionnaire that was created by revising the Fagerström Tolerance Questionnaire (FTQ) (Heatherton *et al.*, 1991). The FTND is shorter and more practical than the FTQ and is the most commonly used test for cigarette addiction. When the two tests were compared, it was suggested that the FTND was preferred because it provided a better assessment than the FTQ and had higher internal consistency and reliability (Payne *et al.*, 1994; Pomerleau *et al.*, 1994; Kozlowski *et al.*, 1994). The FTND consists of six questions and is evaluated by summing the scores of the options that can be given as answers to these questions. A score between zero and two indicates very low addiction; three and four points indicate low addiction; five and six points indicate moderate addiction; seven and eight points indicate high addiction; nine, ten, and eleven points indicate very high addiction.

The FTND provides empirical evidence that addiction levels reflect nicotine intake; for example, some studies (Esen and Arıca, 2018; Al Sharbatti *et al.*, 2022) demonstrate that higher addiction scores are associated with greater nicotine consumption.

### Data analysis

Data analysis of the study was performed using IBM SPSS Version 23.0 (IBM Corp. Released 2015. IBM Statistics for Windows, Version 23.0. Armonk, NY: IBM Corp.). Descriptive statistics are presented as mean ± standard deviation (mean ± ss), median, interquartile range, and minimum and maximum values for continuous variables. Categorical variables were expressed as numbers and percentages. Whether the data conformed to the normal distribution was investigated by Shapiro–Wilk test when n ≤ 50 and Kolmogorov–Smirnov test when n > 50. The homogeneity of variances in pairwise comparisons was evaluated with Levene’s test. Two independent group comparisons were made with the independent sample t test if the assumptions of normality and homogeneity of variances were met, and with the Mann–Whitney U test if the assumption of normality was not met. Comparisons of more than two independent groups were tested with Kruskal–Wallis test. If there was a statistically significant difference between the groups as a result of the Kruskal-Wallis test, the group from which the difference originated was investigated with the Dunn multiple comparison test. Comparisons between categorical variables were evaluated using Pearson’s chi-square test and in cases where the chi-square assumption was not met, the Exact chi-square test was used. The relationships between continuous variables were investigated by Spearman’s rank correlation test. The statistical significance level was p < 0.05.

## Results

There were 146 participants in our study. About 59.6% of the participants were male and 40.4% were female. The age of the youngest participant was 19 years, the age of the oldest participant was 68 years, and the mean age of the participants was 35.8 ± 13.5 years. The sociodemographic data of the participants is given in Table 1.

**Table 1.** Some demographic characteristics of the participants

	n	%
**Gender**		
*Male*	87	59.6
*Female*	59	40.4
**Age**		
*Mean ± SD*	35.8 ± 13.5	
*Min - Max*	19.0 – 68.0	
*Median: (IQR)*	32.5 (24.0)	
**Education Status**		
*Primary school*	7	4.8
*Middle school*	6	4.1
*High school*	37	25.3
*College*	78	53.4
*Master’s/Doctorate*	18	12.3
**Profession**		
*Health worker*	23	15.8
*Office worker*	34	23.3
*Engineer*	1	0.7
*Student*	51	34.9
*Academician*	1	0.7
*Lawyer*	1	0.7
*Other*	35	24.0
**Marital Status**		
*Single/widowed*	83	56.8
*Married*	63	43.2
**Employment Status**		
*Working*	91	62.3
*Not working*	50	34.2
*Retired*	5	3.4
**Body Mass Index**		
*Mean ± SD*	25.6 ± 4.69	
*Min - Max*	16.5 – 46.2	
*Median: (IQR)*	25.6 (6.3)	

Mean: Average, SD: Standard deviation, Min: Minimum, Max: Maximum, IQR: Interquartile range.

When the average exhaled CO levels of the participants were analyzed according to their education levels, the average CO level of the participants with secondary school education was 4.67 ± 3.08, which was the lowest. The average CO level of the participants with primary school education was 13.86 ± 5.46 and the highest. A statistically significant difference was found between the education levels of the participants in terms of CO level (p = 0.002). As a result of the multiple comparison test, a statistically significant difference was found between secondary school and primary school participants in terms of CO level distribution (p = 0.037) and a statistically significant difference was found between college and primary school participants in terms of CO level distribution (p = 0.040).

Looking at the exhaled CO levels of participants from different professions, the mean CO level of students was found to be the lowest and was 5.84 ± 3.61. The mean CO level of the participants whose occupation was office workers was found to be the highest and was 11.65 ± 6.52. A statistically significant difference was found between participants from different professions in terms of CO levels (p < 0.001). As a result of the multiple comparison test, a statistically significant difference was found between students and office workers in terms of CO level (p < 0.001) and a statistically significant difference was found between health workers and office workers in terms of CO level (p = 0.015).

A statistically significant difference was found between single or widowed participants and married participants in terms of exhaled CO level distribution (p < 0.001).

A statistically significant difference was found between non-working and working participants in terms of exhaled CO level distribution (p = 0.001) (Table 2). The distribution of exhaled CO levels according to the demographic characteristics of the participants is summarized in Table 2.

**Table 2.** Distribution of exhaled carbon monoxide (CO) levels according to some demographic characteristics of the participants

	CO levels		p
	Mean ± SD	Median: (IQR)	
**Education Status**			**0.002**^1^
*Primary school*	13.86 ± 5.46	13.00 (5.50)	
*Middle school*	4.67 ± 3.08	3.50 (4.75)	
*High school*	10.19 ± 5.96	9.00 (8.00)	
*College*	7.19 ± 5.32	6.00 (6.75)	
*Master’s/Doctorate*	7.33 ± 6.84	3.50 (8.75)	
**Profession**			**<0.001**^1^
*Health worker*	7.22 ± 6.73	8.00 (7.50)	
*Office worker*	11.65 ± 6.52	12.00 (8.75)	
*Student*	5.84 ± 3.61	6.00 (5.50)	
*Other*	8.82 ± 5.79	8.50 (8.75)	
**Marital Status**			**<0.001**^2^
*Single/widowed*	6.87 ± 5.50	6.00 (6.50)	
*Married*	9.92 ± 5.96	10.00 (9.00)	
**Employment Status**			**0.001**^1^
*Working*	9.67 ± 6.54	9.00 (9.50)	
*Not working*	5.70 ± 3.54	5.50 (5.75)	
*Retired*	6.00 ± 2.45	5.00 (3.00)	

Mean: Average, SD: Standard deviation, IQR: Interquartile range.

1 Kruskal – Wallis test, ^2^Mann – Whitney U test.

A positive, weak relationship was found between age and exhaled CO level, and this relationship was statistically significant (rho = 0.288, p < 0.001) (Table 3).

**Table 3.** Relationship between age and exhaled carbon monoxide (CO) levels

	CO Levels	
	rho	p-value
**Age**	0.288	**<0.001**

rho: Spearman’s rank correlation coefficient.

The lowest measured CO value of the participants was 1.0, the highest was 32.0, and the mean CO value was 8.18 ± 5.88. The lowest percentage of the predicted 6-MWT distance of the participants was 70.0, the highest percentage of the predicted 6-MWT distance was 117.0, and the mean percentage of the predicted 6-MWT distance was 90.7 ± 7.91. Half of the participants had an addiction score below 4.0, and the mean addiction score was 3.81 ± 2.60. About 34.2% of the participants were very mildly dependent, 27.4% were mildly dependent, 13.0% were moderately dependent, 15.8% were severely dependent, and 9.6% were severely dependent (Table 4).

**Table 4.** Distribution of aerobic capacity, exhaled carbon monoxide (CO) level, addiction score, and degree of addiction

	n	%
**CO Measurement**		
Mean ± SD	8.18 ± 5.88	
Min - Max	1.00–32.0	
Median (IQR)	8.18 (7.00)	
**6 MWT, %pred**		
Mean ± SD	90.7 ± 7.91	
Min - Max	70.0–117.0	
Median (IQR)	90.3 (10.4)	
**6-MWT (m)**		
Mean ± SD	579.1 ± 65.4	
Min - Max	411.2–750.2	
Median (IQR)	581.5 (75.5)	
**Dependency Score**		
Mean ± SD	3.81 ± 2.60	
Median (IQR)	4.00 (3.75)	
**Dependency Rating**		
*Very Low*	50	34.2
*Low*	40	27.4
*Middle*	19	13.0
*Advanced*	23	15.8
*Very Advanced*	14	9.6

Mean: Average, SD: Standard deviation, Min: Minimum, Max: Maximum, 6-MWT: 6-minute walk test, IQR: Interquartile range.

A negative, very weak relationship was found between the percentage of the predicted 6-MWT distance and exhaled CO level, and this relationship was not statistically significant (rho = −0.112, p = 0.177). A positive, moderate relationship was found between addiction score and exhaled CO level, and this relationship was statistically significant (rho = 0.483, p < 0.001) (Table 5).

**Table 5.** Association of aerobic capacity and addiction score with exhaled carbon monoxide (CO) levels

	CO Level	
	rho	p-value
**6-MWT, %pred**	−0.112	0.177
**Dependency Score**	0.483	<0.001

6-MWT: 6-minute walk test, rho: Spearman’s rank correlation coefficient.

## Discussion

In this study, exhaled CO levels were found to have a significant positive association with nicotine dependence and a negative association with aerobic capacity, although not statistically significant.

A statistically significant difference was found between the education levels of the participants in terms of exhaled CO level (p = 0.002). As a result of the multiple comparison test, p = 0.037 was found between the participants with secondary school and primary school education, p = 0.040 was found between the participants with college and primary school education, and these differences were found to be statistically significant in terms of CO level distribution, more in participants with primary school graduates. In the study conducted by Zhang et al., it was observed that the CO value decreased as the education level increased in men and it is like our study (Zhang *et al.*, 2013).

A statistically significant difference was found between participants from different professions in terms of exhaled CO levels (p < 0.001). As a result of the multiple comparison test, a statistically significant difference was found between students and office workers with p < 0.001 and between health workers and office workers with p = 0.015, with a higher CO level in office workers. In the literature review, no study examining the relationship between occupation and exhaled CO was found; however, among the studies on smoking status and occupations, Ünal and Marakoğlu found a statistically significant difference with a higher rate of smoking in workers and office workers, and the high rate of smoking in office workers was similar to our study (p < 0.001) (Unal and Marakoğlu, 2020). It is thought that the high smoking rates and exhaled CO levels in office workers may be due to socioeconomic reasons.

A statistically significant difference was found between single or widowed participants and married participants in terms of exhaled CO level distribution, with a higher level in married participants (p < 0.001). In the literature review, no study examining the relationship between marital status and exhaled CO was found, but among the studies on smoking status and marital status, Wan Puteh et al. found a statistically significant difference with a higher rate of smoking in married participants, which is like our study (p = 0.047) (Wan Puteh *et al.*, 2023). It is thought that the high exhaled CO level and smoking rates in married people may be related to stress and family livelihood problems.

A statistically significant difference was found between non-working participants and working participants in terms of exhaled CO level distribution, with a higher difference in working participants (p = 0.001). In the literature review, no study examining the relationship between employment status and exhaled CO level was found. It is thought that higher exhaled CO levels in employees may be due to smoking more due to work stress. In contrast to our study, Bilgiç et al. reported that unemployed people may be more likely to smoke because they can find opportunities more easily (Bilgiç *et al.*, 2010).

A positive, weak correlation was found between age and exhaled CO level and this correlation was statistically significant (rho = 0.288, p < 0.001). Sönmez et al. found a statistically significant but negative correlation between age and exhaled CO level (r = −0.145 p ≤ 0.001) (Sönmez *et al.*, 2017). It is thought that this may be due to the differences in the samples of the studies. In the study conducted by Pan et al., a weak positive correlation was found between age and exhaled CO level, which is similar to our study (p = 0.05) (Pan *et al.*, 2021). This may suggest a more interestingly associated relationship between CO levels and sociodemographic factors such as professional groups, marital status, and educational level that might require further exploratory studies beyond what could be given in the study.

In our study, the lowest exhaled CO value measured among the participants was 1.0 and the highest was 32.0. Half of the participants had a CO measurement above 8.18 and the mean CO measurement was 8.18 ± 5.88. In the study conducted by Demirbaş and Kutlu, the mean CO level of smokers was found to be 12.22 ± 5.87, which is higher than our study (Demirbaş and Kutlu, 2018). This is thought to be because the number of cigarettes smoked in their study was higher, or the CO measuring device was a different brand from the device we used, or the measurement was performed immediately after smoking.

We found a positive correlation between addiction score and exhaled CO levels, statistically significant in our study group, with rho = 0.483, p < 0.001. Moreover, the differences of CO levels among addiction classes were statistically significant, with p < 0.001, especially between the very mildly addicted group and all the other groups. These findings are in accordance with the previous literature. In the study conducted by Esen and Arıca, a positive moderate correlation was obtained between addiction score and CO levels, with r = 0.338 and p = 0.001 (Esen and Arıca, 2018). Sharbatti et al. reported higher levels of CO among people with moderate to high nicotine dependence as opposed to the low-dependent group, and it was statistically different at p = 0.001. For every unit increase in CO, nicotine dependence increased 10% more (aOR = 1.1, 95% CI: 1.0–1.2, p = 0.025) (Al Sharbatti *et al.*, 2022). In this context, Sönmez et al. reported a positive correlation, with r = 0.293 and p < 0.001 (Sönmez *et al.*, 2017). Other studies like that of Sugavanesh and Pushpanjali (r = 0.374, p < 0.001), Guan and Ann, (p= 0.01), and Vançelik et al. (r = 0.411, p < 0.001) have further supported it (Sugavanesh and Pushpanjali, 2018; Guan and Ann, 2012; Vançelik *et al.*, 2009). There were no conflicting findings among the literature reviewed.

Our study revealed that participants’ aerobic capacity was largely unaffected, and there was only a weak, non-significant correlation between aerobic capacity and exhaled CO levels. Similarly, Camarri et al. found no significant link between aerobic capacity, measured by the 6-MWT, and exhaled CO levels (Camarri *et al.*, 2006). Other research, such as studies by Mesquita et al. and Furlanetto et al., indicated that older smokers showed reduced aerobic capacity compared to non-smokers (Mesquita *et al.*, 2015; Furlanetto *et al.*, 2014). The younger average age and lower body mass index (BMI) of participants in our study may explain the difference in results, as these factors are associated with less smoking exposure and better aerobic performance (Radovanovic *et al.*, 2014). In addition, the lack of assessment of CO levels both before and after exercise could have influenced the weak correlation observed in our findings.

In the literature, studies evaluating the relationship between the FTND score and exhaled CO level have been found, but no study has been found to examine the effect of physical activity on exhaled CO. Using the 6-MWT, which is one of the direct observation methods instead of questionnaire methods when evaluating physical activity, more objective evaluation and using an electronic device that measures exhaled CO value in ppm enabled us to obtain more reliable results. The fact that the 6-MWT was administered by the same physiotherapist, exhaled CO measurement, FTND and questionnaire forms were administered by the same researcher and all data were obtained through a face-to-face study made it more reliable.

Our study is a cross-sectional study. The limitations of our study include the fact that it was conducted in a single centre and in a small sample group and the limited age range. There were 7 people who did not want to participate in the study because the study included a walking test, and they thought it was time consuming or because they did not find their current clothing and shoes suitable. No participants left the study after starting it. In our study, the desire to quit smoking was not questioned. Before exhaled CO measurements were performed, the duration of last smoking and how much time had passed since the last cigarette were not questioned. We measured physical activity capacity in our study, but additional tests measuring lung capacity may make the study results more meaningful. It should also be recognized that future studies may provide a more comprehensive perspective on the interpretation of CO levels by addressing passive smoking.

## Conclusion

In the light of the data obtained in our study, we found that the level of addiction evaluated with the FTND had a positive moderate statistically significant effect on exhaled CO level. Although we found that the physical activity capacity (aerobic capacity) evaluated with the 6-MWT had a very weak negative relationship on exhaled CO level, it was not statistically significant. However, we think that the significance rate will increase with multi-centre studies with more participants. We found that the studies investigating the effect of physical activity capacity on exhaled CO level in the literature are very limited and we believe that more studies are needed in this regard. Primary care, especially from family physicians, is essential for identifying smoking addiction in its early stages and helping people quit smoking and adopt better lifestyles. Physician advice to quit smoking, which often comes too late when health has already deteriorated, is a necessary but insufficient component of intervention. Early and culturally tailored advice is important to address smoking at the societal level.
